# Supplementary figures and images for: Does receiving a SARS-CoV-2 antibody test result change COVID-19 protective behaviors? Testing risk compensation in undergraduate students with a randomized controlled trial
Source: PLoS One. 2022 Dec 20;17(12):e0279347. doi: 10.1371/journal.pone.0279347 (PMC9767325; doi:10.1371/journal.pone.0279347)

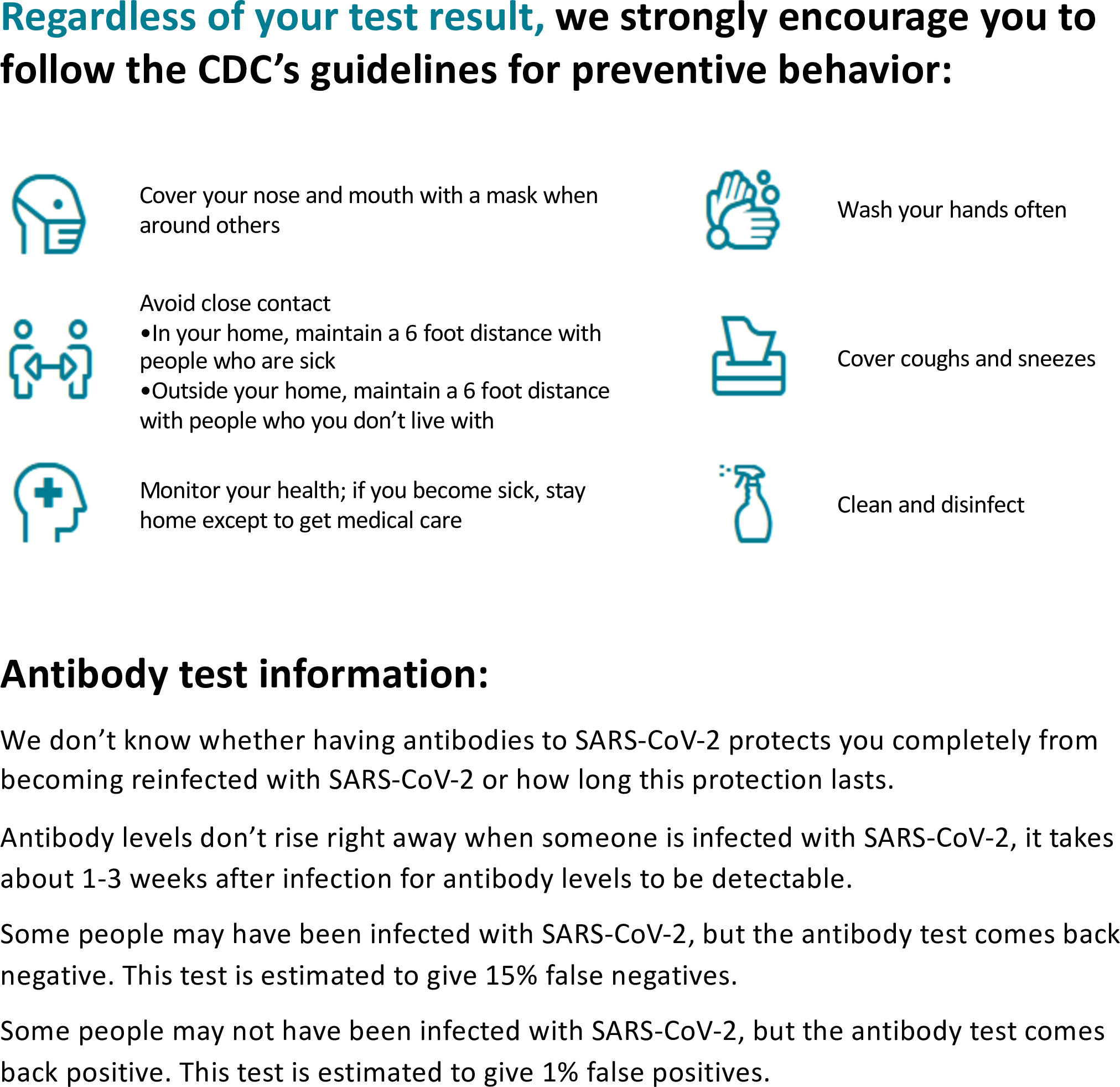

Supplement: S1 Fig — (TIF) [file pone.0279347.s001.tif]

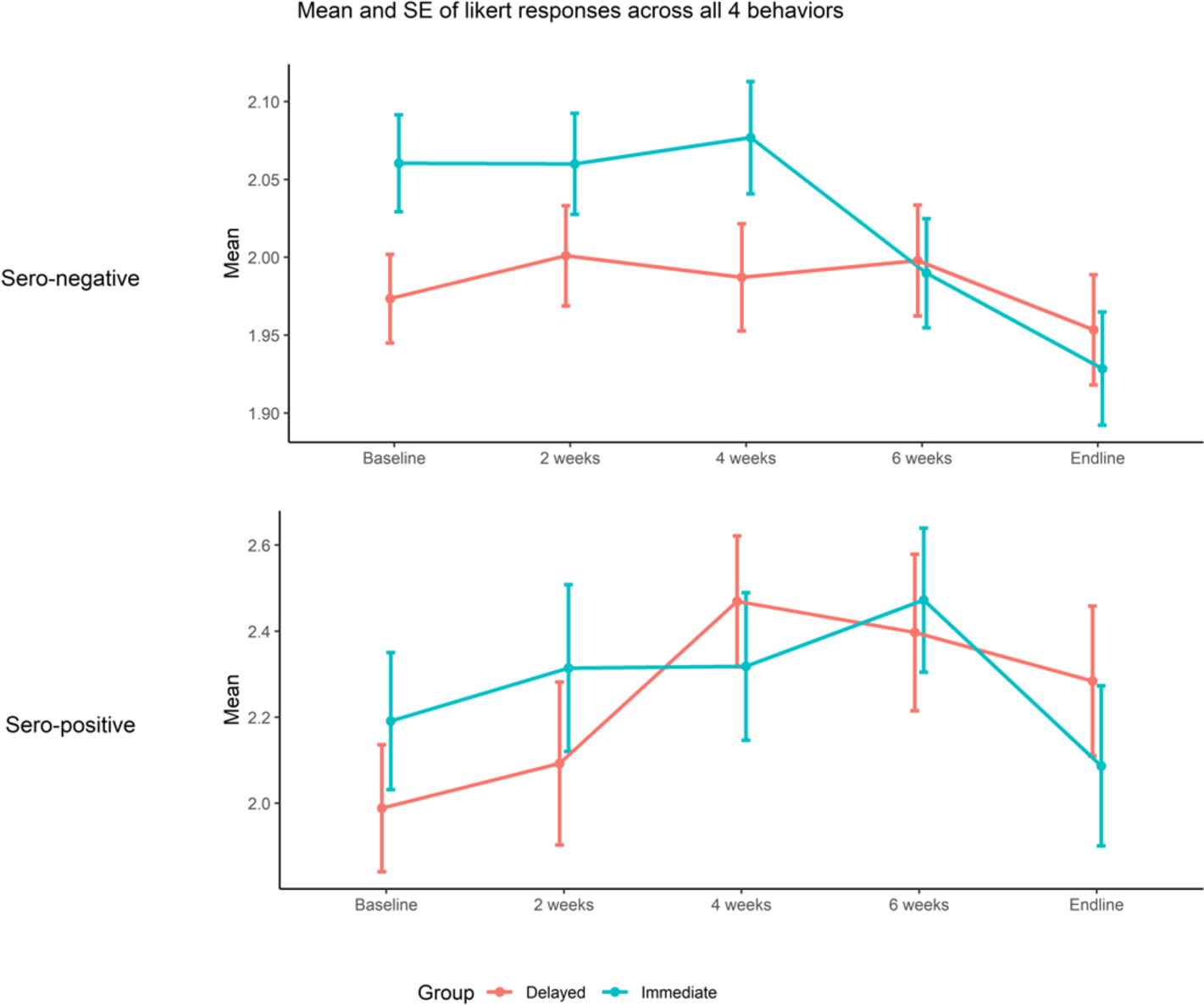

Supplement: S2 Fig — (TIF) [file pone.0279347.s002.tif]
