# Supplementary material for: Does receiving a SARS-CoV-2 antibody test result change COVID-19 protective behaviors? Testing risk compensation in undergraduate students with a randomized controlled trial
Source: PLoS One. 2022 Dec 20;17(12):e0279347. doi: 10.1371/journal.pone.0279347 (PMC9767325; doi:10.1371/journal.pone.0279347)
Supplement: S1 Table — (DOCX) [file pone.0279347.s003.docx]

| **S1 Table.**Sensitivity analysis for association between treatment condition (immediate vs delayed antibody test results) and mean frequency of engagement in protective behaviors at 2 weeks, stratified by baseline antibody status | | | | |
| --- | --- | --- | --- | --- |
| **Variable** | ***B* (95% CI)** | ***t*** | ***p*** |  |
| *Negative antibody test results (n=944)* | | | | |
| Treatment condition | 0.06 (-0.03, 0.15) | 1.29 | 0.197 |  |
| R^2^=0.0018, F(1, 942) = 1.66, *p* = 0.197 | | | | |
| *Positive antibody test results* *(n=40)* | | | | |
| Treatment condition | 0.22 (-0.33, 0.78) | 0.81 | 0.424 |  |
| R^2^=0.017, F(1, 38) = 0.65, *p* = 0.424 | | | | |
