# Supplementary material for: Does receiving a SARS-CoV-2 antibody test result change COVID-19 protective behaviors? Testing risk compensation in undergraduate students with a randomized controlled trial
Source: PLoS One. 2022 Dec 20;17(12):e0279347. doi: 10.1371/journal.pone.0279347 (PMC9767325; doi:10.1371/journal.pone.0279347)
Supplement: S1 Data — (DOCX) [file pone.0279347.s006.docx]

# **Longitudinal SARS-CoV-2 antibody testing in IU undergraduate students**

## **Molly Rosenberg, Assistant Professor**

## **Department of Epidemiology and Biostatistics**

## **Indiana University School of Public Health-Bloomington**

**Investigative Team:**

Christina Ludema, Assistant Professor

Department of Epidemiology and Biostatistics

Indiana University School of Public Health-Bloomington

Jon Macy, Associate Professor

Department of Applied Health Science

Indiana University School of Public Health-Bloomington

David Allison, Dean

Indiana University School of Public Health-Bloomington

Don D. Dumayas, Doctoral Student

Department of Applied Health Science

Indiana University School of Public Health-Bloomington

**Support Provided by:**

Donations from 4 private donors to the IU Foundation

**Table of Contents:**

**Study Schema**

**1.0** **Background & Rationale**

**2.0** **Objective(s)**

**2.1** **Primary Objective**

**2.2** **Secondary Objective**

**2.3** **Tertiary/Exploratory/Correlative Objectives**

**3.0** **Outcome Measures**

**3.1** **Primary Outcome Measures**

**3.2** **Secondary Outcome Measures**

**3.3** **Tertiary/ Exploratory/ Correlative Outcome Measures**

**4.0** **Eligibility Criteria**

**4.1** **Inclusion Criteria**

**4.2** **Exclusion Criteria**

**5.0** **Study Design**

**6.0** **Enrollment/Randomization**

**7.0** **Study Procedures**

**8.0** **Study Calendar**

**9.0** **Reportable Events**

**10.0** **Data Safety Monitoring**

**11.0** **Study Withdrawal/Discontinuation**

**12.0** **Statistical Considerations**

**13.0** **Data Management**

**14.0** **Privacy/Confidentiality Issues**

**15.0** **Follow-up and Record Retention**

**16.0** **References**

**17.0** **Appendix**

**1.0** **Background & Rationale**

Our primary goal for this study to assess whether receiving the results of an antibody test changes protective behavior to avoid SARS-CoV-2 infections (i.e., mask-wearing, physical distancing, limiting close contacts/avoiding crowds, hand-washing, avoiding contact with high-risk individuals). While studies have been published on the cross-sectional relationship between risk perception and other demographic characteristics and health behaviors that are protective for SARS-CoV-2 infection (see citations), to our knowledge there have been no studies showing the effect of receiving information about antibody positivity on protective behavior. Not only can results from this study be used to better model transmission, a better understanding of college student’s risk perception around SARS-CoV-2 infections has implications for future vaccination strategies as well. There are concerns that a desire to return to “normal” life in combination with reduced perception of risk could have negative consequences for uptake of vaccination (Johns Hopkins Center for Health Security 2020 report, *The Public’s Role in COVID-19 Vaccination: Planning Recommendations Informed by Design Thinking and the Social, Behavioral, and Communication Sciences*).

The antibody test we will use is named ‘SARS-CoV-2 IgM/IgG rapid assay kit (Colloidal Gold)’. It provides a fast, on-site, and accurate detection of IgM/IgG antibodies against SARS-CoV-2, with positive results of IgM antibodies indicating a recent infection, while positive results of IgG antibodies signaling a longer or previous infection. It can detect IgM and IgG antibodies against SARS-CoV-2 in human specimens of serum, plasma, or venous whole blood.

**2.0** **Objective(s)**

**2.1** **Primary Objective:** To assess whether receiving the results of an antibody test changes protective behavior to avoid SARS-CoV-2 infections

**2.2** **Secondary Objective:** To assess the relationship between alcohol consumption and drinking behaviors with incidence of new SARS-CoV-2 infections on a college campus

**2.3** **Tertiary Objectives:**

1. To assess the relationship between vaping and incidence of new SARS-CoV-2 infections on a college campus
2. To assess the behavioral and demographic correlates of antibody positivity at baseline

**3.0** **Outcome Measures/Endpoints**

**3.1** **Primary Outcome Measures:** Protective behaviors against SARS-CoV-2: 1) compliance with mask wearing guidance; 2) compliance with physical distancing guidance; 3) limiting close contacts/avoiding crowds; 4) hand-washing; 5) avoiding contact with high-risk individuals. Each of these outcomes will be self-reported by participants in a web-based survey every 2 weeks during study follow-up.

**3.2** **Secondary Outcome Measures:** SARS-CoV-2 seroconversion: This outcome will be assessed by comparing SARS-CoV-2 serostatus at baseline (September 2020) to endline (November 2020). Those who were antibody negative at baseline but antibody positive at endline will be considered seroconverters in this study.

**4.0** **Eligibility Criteria**

**4.1** **Inclusion Criteria**

1. Age 18 years or older
2. Current IU undergraduate student
3. Current resident of Monroe County, Indiana

**4.2** **Exclusion Criteria**

1. Younger than 18 years old
2. Current residence outside of Monroe County, Indiana

**5.0** **Study Design**

Our approach to answering this question is to randomly sample n=1,700 undergraduate students from Indiana University. We will administer a rapid antibody test and will randomize participants into two groups, stratified by the test result (i.e., antibody test positive or antibody test negative, see Figure 1). One group will receive their test results within a day of the test, while the other group will receive the test results after a four-week waiting period. We will assess frequency of protective behaviors in bi-weekly web-based behavioral surveys.

| 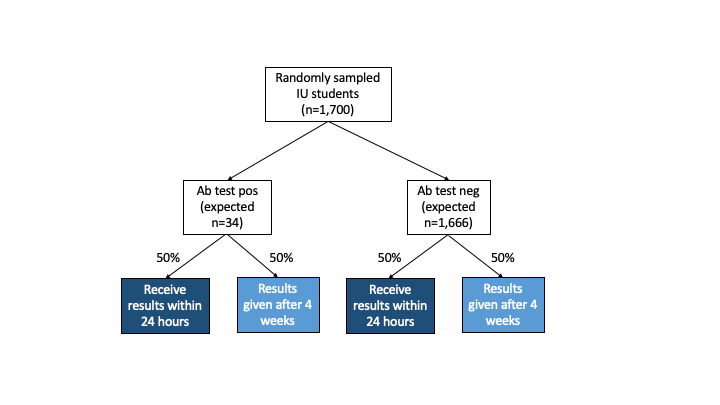 | **Figure 1A.** Schematic of randomization plan |
| --- | --- |
| 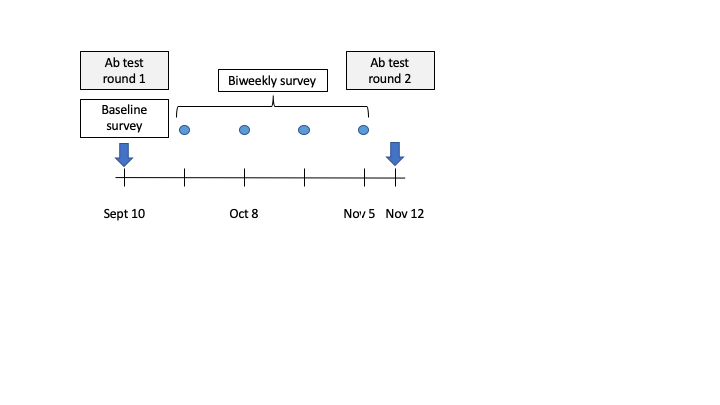 | **Figure 1B.** Timeline of data collection |

**6.0** **Enrollment/Randomization**

***Identification and recruitment***

We will randomly sample IU undergraduate students from the full IU undergraduate student population. The random sample and their contact information will be provided by the IU Institutional Research and Decision Support team. The desired sample size for the study is 1700 students. To account for 30% refusal and ineligibility, we will initially request a random sample of 2430 students. A refreshed sample will be requested if refusals or ineligibility rates are higher than anticipated.

We will contact each of the sampled potential participants with a recruitment email. This email will contain information about the study and will include a link that potential participants can click on to receive further information about the study and conduct a brief screening to assess eligibility. If the potential participant is eligible and willing to participate, they will navigate to provide written informed consent, and schedule their baseline laboratory visit. If the participant does not respond to the initial recruitment email, up to 3 follow-up reminder emails will be sent. These follow-up reminder emails will also include links to provide study information and informed consent.

***Informed consent and enrollment***

Information about the study and participants rights will be presented in writing in a web-based document navigated to from the initial recruitment email and follow-up emails. Our informed consent protocol will include the contact information for study staff in the web-based informed consent documents so that potential participants can ask any questions they may have outstanding about the study and/or their rights.

Participation in the study is completely voluntary and every effort will be made to ensure that potential participants understand the risks and benefits of participation as well as what will be asked of them if they decide to participate. Accordingly, they may take as much time as needed to peruse the informed consent documents before digitally signing. Additionally, research staff will be available by phone and email to answer any questions that the potential participant might have about the study and their participation in it.

The informed consent documents provide information about the study, including the purpose of the study, the study procedures, risks associated with participation, compensation arrangement, and contact numbers for the principal investigators. Potential participants will have to sign the informed consent before being enrolled in the study, accessing the on-line baseline survey, and scheduling the baseline laboratory testing. Participants will sign an web-based consent form. Digital copies of the informed consent documents will be kept in a secure Box Health folder only accessible to study personnel.

**7.0** **Study Procedures**

This study will take place between September 10-November 18, 2020, and will ask participants to participate in: a web-based baseline survey, two rounds of SARS-CoV-2 serological testing (September and November), and bi-weekly web-based behavioral surveys (4 total surveys). Each is described in more detail below:

*Web-based baseline survey:* The baseline survey is designed to collect data on participant demographics, SARS-CoV-2 protective behaviors, alcohol drinking habits, nicotine use, and personality profile and should take less than 30 minutes to fill out. Participants who provide informed consent will be provided the link to the survey which they can fill out at a time convenient to them prior to first round of serological testing.

*SARS-CoV-2 serological testing:* There will be two rounds of SARS-CoV-2 serological testing, once at baseline (September 10-13) and once at endline (November 15-18). The serological testing visits will involve in-person laboratory testing for SARS-CoV-2 antibodies. The laboratory test involves a fingerstick to provide a small blood sample for the antibody test kits. In-person laboratory tests will be conducted in an outdoor location outside of IU SPH (tennis courts). Should inclement weather necessitate an indoor location, an alternate setting (SPH gymnasium) will be secured. Parking will be paid for subjects who need to drive to this location.

Antibody test results will eventually be provided to all study participants via secure link sent by email. With this message, we will include a clearly written information sheet about the chance for inaccurate test results and how it is still unknown whether previous infections confer immunity to future infections. We will clearly counsel participants to not use the results of the tests as proof of a previous SARS-CoV-2 infection, nor as a reason to change their behaviors. If participants wish to participate in the study, but do not wish to be provided with their antibody test results, they will be able to opt out of the results provision. If any participants opt out of receiving their test results, these participants would essentially create a third category of respondents: those who do not receive their results at all. However, these participants will still be analyzed with the trial arm to which they were randomized (intent to treat analysis).

The primary experiment will be assessing whether provision of the antibody test results leads to behavior change with respect to personal protective behaviors. To that end, we will randomize all participants to a trial arm that immediately receive results (within 24 hours) or a trial arm with a delayed provision of results (after 4 weeks). So as to not incentivize early drop-out, if a participant in the delayed results arm drops out early, they will still be provided their test results at the regularly scheduled time, not earlier. All other procedures between arms are identical. The endline laboratory test results will be delivered to all participants in the same timeframe – within 24-72 hours. The early and delayed test result intervention will have completed after the first round of testing.

*Web-based behavioral surveys:* To assess whether or not the provision of antibody test results changes behaviors, we will query self-reported behaviors in a short web-based survey every two weeks under observation. Links to these surveys will be sent to participants at regular bi-weekly intervals. Participants will fill out these short follow-up surveys on their computers or mobile devices. They are designed to take about 5 minutes or less to complete for each survey.

*Compensation:* We will use a tiered compensation scheme to incentivize the sustained participation necessary for this study. Each participant will be eligible to receive compensation of $30 total value in cash or gift cards if they complete all study procedures. Completion of the baseline survey and baseline serological testing procedures can earn participants $10. Completion of each of the four web-based behavioral surveys can earn participants $3 for a total of $12 possible compensation. Finally, completion of the endline serological testing can earn participants an additional $8. The total possible value of compensation for a participant completing all study procedures is $30, with incrementally lower compensation values for those with incomplete study procedures. All compensation will be delivered to participants at the end of the study follow-up period.

**8.0** **Study Calendar**

The table below provides estimated timing of all proposed study procedures. Variations of +/- 7 days from the scheduled visit are permitted.

|  | Baseline | | Follow-up | | | | Endline |
| --- | --- | --- | --- | --- | --- | --- | --- |
|  | Day 0 | Day 1 | Wk 2 | Wk 4 | Wk 6 | Wk 8 | Wk 9 |
| **STUDY PROCEDURES** |  |  |  |  |  |  |  |
| Screening |  |  |  |  |  |  |  |
| Baseline survey |  |  |  |  |  |  |  |
| SARS-CoV-2 antibody test |  |  |  |  |  |  |  |
| Provision of results to ‘Immediate’ arm |  |  |  |  |  |  |  |
| Provision of results to ‘Delayed’ arm |  |  |  |  |  |  |  |
| Behavioral survey |  |  |  |  |  |  |  |

**9.0** **Reportable Events**

The study team does not anticipate that any of the study activities will cause adverse events. However, in case of a reportable safety concern, contact numbers for the principal investigators will be included in study documents, including an office phone number and an after-hours phone number, in case of any time-sensitive safety concerns. Study staff will promptly report any unanticipated adverse events related to (or possibly related to) study participation that suggests that study participants are at greater risk of harm than previously anticipated. This passive collection of data on adverse events will begin at the time of consent.

**10.0** **Data Safety Monitoring**

Data will be collected and stored in REDCap databases and transferred to a secure Box Health folder for analysis and for sharing with approved study team members. Only approved study staff will have access to the data. PI Molly Rosenberg will lead the data safety and monitoring. She will be responsible for monitoring data quality, subject recruitment, accrual, retention, outcome and adverse event data, and procedures designed to protect the privacy of subjects. Stephanie Dickinson at the IU SPH-B Biostatistics Consulting Center will assist with data and safety monitoring. Her role is as a biostatistician consultant and she operates independently from the larger study team. Both Rosenberg and Dickinson will independently monitor the data at the recruitment stage and halfway through the study period (Week 4). At the Week 4 mid-study monitoring, Rosenberg and Dickinson will assess for differences between study arms for pre-specified behavioral outcomes and unanticipated adverse events. In the unlikely event that adverse events are reported at statistically significant higher levels in either arm, we will transparently share this information with all study participants. No pre-planned stopping rules will be in place as both arms will have received their test results by the halfway point of the study. The additional study procedures following the halfway point of the study is observational longitudinal follow-up; there are no additional interventions.

**11.0** **Study Withdrawal/Discontinuation**

If a participant decides to withdraw from the study, they simply need to call any research staff member or one of the principal investigators and inform the research staff member of their decision. These contact numbers will be listed in the informed consent and study information documents. Once they express that they would like to be withdrawn from the study, they will be immediately removed from the study. They will receive the dollar amount compensation up to when they withdrew according to the previously described payment arrangement.

**12.0** **Statistical Considerations**

At n=1700 sample size, this study will be well-powered to be able to detect even relatively small behavior changes between the study arms (immediate vs. delayed provision of antibody test results). We ran power calculations for the subpopulation of participants who we assume will have negative antibody test results (n=1665) and assuming 20% loss to follow up (n=1326). At this effective sample size, we parameterized our power calculation at alpha=0.05 to assess the relative difference in frequencies of dichotomous outcomes ranging from rare (10% background rate), to fairly common (40% background rate), to very common (80% background rate). For rare outcomes, our minimum detectable effect at 80% power is an odds ratio of 1.60. For fairly common outcomes, our minimum detectable effect at 80% power is an odds ratio of 1.37. For very common outcomes, our minimum detectable effect at 80% power is an odds ratio of 1.51.

For the first objective assessing whether reported protective behaviors are different between study arms (immediate vs. delayed test results provision), we will use log-binomial regression models to compare the dichotomous outcomes, and log-linear regression models to compare continuous outcomes between groups.

For the more exploratory secondary and tertiary objectives, we will use log-binomial regression models to assess whether the following exposures are associated with risk of SARS-CoV-2 sero-conversion over the 9 weeks of follow-up:

-Alcohol consumption (frequency and magnitude)

-Attendance at social events involving alcohol consumption

-E-cigarette use (frequency and magnitude)

Missing data: We anticipate loss to follow-up to be around 20% in our study. Participants may also contribute missing data if they have incomplete study procedures, miss survey rounds, or miss individual survey questions. We intend to assess the magnitude and patterns of missing data in our study critically. If appropriate, we will conduct multiple imputation to impute values for these missing datapoints. We will also conduct sensitivity analyses to understand the potential influence of missing data for our results and transparently report these findings.

**13.0** **Statistical Data Management**

Survey data (baseline and follow-up surveys) will be collected via web-based surveys and stored electronically in REDCap. Antibody test result data will be entered by study staff at time of test into an electronic REDCap data collection form and similarly stored on the secure REDCap server. The longer-term storage site for the datasets will be in a secure Box Health folder so that access can be granted to study team members, and analyses can be conducted. These datasets will be combined for the statistical analysis part of the study, merged on a common study ID assigned to each participant at baseline. The study ID will be an alpha numeric key with no identifiers. The link between identifying participant data and study ID will be maintained in a separate secure dataset on a Box Health folder, only accessible to study staff as needed to communicate with participants and schedule visits. Data will be regularly checked and cleaned for quality control (unusual patterns of data, outliers, etc).

**14.0** **Privacy/Confidentiality Issues**

Rigorous efforts will be made to keep the personal information obtained in the study confidential. Although the study team will do everything possible to protect participant information, we cannot guarantee absolute confidentiality. To minimize the risk of breach of confidentiality, we will use secure software to collect and store the data. The survey data and antibody test results will be collected and stored using the REDCap platform and servers. The REDCap platform has strong data security (see: <https://kb.iu.edu/d/bddn> ). Additionally, for analysis, the merged dataset will be stored securely in a password-protected remote Box Health Data folder, which is designed with extra protections for protected health information (PHI) (<https://kb.iu.edu/d/bfrt>). Participant physical privacy will be protected as the surveys will be conducted remotely on the internet. Accordingly, participants may choose to take the surveys when and where they wish and can choose a private location to do so if they prefer.

Finally, we plan to share antibody test results with each individual participant via a secure link sent by email. We will set up several precautions so that participants’ private test results are protected:

1) The email message itself will not contain the test result.

2) Participants can opt out of receiving their test results via email.

**15.0** **Follow-up and Record Retention**

Informed consent documents will be collected electronically via REDCap and stored electronically as pdfs on a secure Box Health server. They will be kept for a minimum of 3 years after the termination of the study. After 3 years, the informed consent documents will be deleted. All study data will be kept securely in the Box Health folder indefinitely and may be used in future work. However, no identifying information will be revealed in any published work and only authorized research staff will have access to the data. This will be limited to the principal investigators, trained staff/students entering or checking the quality of the data, and trained staff/students using the data to conduct the statistical analyses proposed in this study or in future, similar research.

**16.0** **References**

1. Bruine de Bruin W, Bennett D. Relationships Between Initial COVID-19 Risk Perceptions and Protective Health Behaviors: A National Survey. *Am J Prev Med*. 2020;59(2):157-167. doi:10.1016/j.amepre.2020.05.001

2. Seale H, Heywood AE, Leask J, et al. COVID-19 is rapidly changing: Examining public perceptions and behaviors in response to this evolving pandemic. *PLoS One*. 2020;15(6):e0235112. Published 2020 Jun 23. doi:10.1371/journal.pone.0235112

3. Clements JM. Knowledge and Behaviors Toward COVID-19 Among US Residents During the Early Days of the Pandemic: Cross-Sectional Online Questionnaire. *JMIR Public Health Surveill*. 2020;6(2):e19161. Published 2020 May 8. doi:10.2196/19161

**17.0** **Appendix**

Please see attached appendices:

1. Antibody test package insert
2. Baseline questionnaire
3. Follow-up questionnaire
